# Implementation of Gender Identity and Assigned Sex at Birth Data Collection in Electronic Health Records: Where Are We Now?

**DOI:** 10.3390/ijerph18126599

**Published:** 2021-06-19

**Authors:** Hale M. Thompson, Clair A. Kronk, Ketzel Feasley, Paul Pachwicewicz, Niranjan S. Karnik

**Affiliations:** 1 Department of Psychiatry and Behavioral Science, Rush University Medical Center, Chicago, IL 60612, USA; ketzel_feasley@rush.edu (K.F.); paul_pachwicewicz@rush.edu (P.P.); niranjan_karnik@rush.edu (N.S.K.); 2 Department of Biomedical Informatics, University of Cincinnati College of Medicine, Cincinnati, OH 45267, USA; kronkcj@mail.uc.edu

**Keywords:** SO/GI data, nonbinary, population health, clinical informatics, transgender health disparities

## Abstract

In 2015, the United States Department of Health and Human Services instantiated rules mandating the inclusion of sexual orientation and gender identity (SO/GI) data fields for systems certified under Stage 3 of the Meaningful Use of Electronic Health Records (EHR) program. To date, no published assessments have benchmarked implementation penetration and data quality. To establish a benchmark for a U.S. health system collection of gender identity and sex assigned at birth, we analyzed one urban academic health center’s EHR data; specifically, the records of patients with unplanned hospital admissions during 2020 (N = 49,314). Approximately one-quarter of patient records included gender identity data, and one percent of them indicated a transgender or nonbinary (TGNB) status. Data quality checks suggested limited provider literacy around gender identity as well as limited provider and patient comfort levels with gender identity disclosures. Improvements are needed in both provider and patient literacy and comfort around gender identity in clinical settings. To include TGNB populations in informatics-based research, additional novel approaches, such as natural language processing, may be needed for more comprehensive and representative TGNB cohort discovery. Community and stakeholder engagement around gender identity data collection and health research will likely improve these implementation efforts.

## 1. Introduction

### 1.1. Who Counts as Transgender? What Counts as Sexual Orientation? The Case of Patient Doe

In a 2020 quality improvement report to determine the prevalence of hospital patient electronic health records (EHR) that include gender identity (GI) and sexual orientation (SO) data, 41 patients had a sexual orientation listed as “transgender” (*n* = 13) or “neither exclusively man nor woman” (*n* = 28). A quality check of one such patient’s record, patient “Doe”, indicated a possible mapping error of a gender identity noted by a provider in the social history, instead of the sexual orientation that was listed in the demographics table. Although the report indicated a “transgender/male-to-female” sexual orientation, patient Doe’s demographic SO/GI tab read: male (assigned sex at birth), male (gender identity), heterosexual (sexual orientation), and had no pronouns indicated. Doe is referred to with he/him pronouns throughout his record. In the social history section, however, where the institution had been collecting SO/GI data prior to 2020, a note reads that patient Doe had seen a physician about “transgender reassignment, male-to-female.” Doe’s photo in the patient snapshot appears very typically “male” in presentation, and the photo of Doe’s driver’s license shows “sex: M.” Notably, the surgical history section dating back years, reads “transgender s/p M to F reassignment surgery,” and medication notes through 2021 indicate the use of estrogen.

### 1.2. Background and Significance

Little is known about the quality of electronic health record data regarding gender identity (GI) and sexual orientation (SO). From Doe’s case, we can deduce that the “transgender” sexual orientation reflects a mapping error since his actual record’s SO data field indicates heterosexual. But how should one count this patient in terms of gender identity? In our quality improvement analysis that follows, patient Doe is counted as cisgender, and it appears that is how he wants to be recognized in the clinical context. Doe’s structured data fields for legal sex, assigned sex at birth (ASAB), and GI are aligned as male, but keywords in the social history and clinical notes of the EHR, indicate the presence of gender-confirming surgical and hormonal interventions from an outside provider, leading us to interpret patient Doe as a transgender person who has medically transitioned but has not legally or entirely socially transitioned. EHRs have purposes that extend beyond the patient in the clinical context. Increasingly, EHR data are the basis for a range of health research, and in the context of research and equity, perhaps we would want to count transgender patients, such as Doe, who are not “out” in clinical settings where they may not feel comfortable or safe. This case provides insight into the uses, misuses, and the importance of SO/GI EHR data collection while also extending the question of who counts as transgender and, increasingly, nonbinary [1,2,3,4,5].

Formulations of gender identities (see for example, [6,7,8]) and modalities [9] beyond U.S. borders have been contested and redefined over time at local levels across the globe. International organizations like the United Nations (UNESCO), World Professional Association of Transgender Health (WPATH), and the World Health Organization’s (WHO) International Classification of Diseases (ICD) have made more sweeping attempts at the establishment of global definitions moving from, for example, an individual pathology (i.e., Gender Identity Disorder in ICD-9) to a more socially circumscribed illness (i.e., Gender Dysphoria in ICD-10) to a relatively neutral status (i.e., Gender Incongruence coming in ICD-11) [10,11,12]. Both WPATH and UNESCO have established more flexible definitions for transgender and nonbinary (TGNB) statuses, essentially establishing TGNB as any gender that differs from the one associated with one’s sex assigned at birth [13]. This broad definition does not negate the complexities of gender across and within nations, cultures, histories, and healthcare systems, and reflects a growing prevalence and/or awareness of nonbinary identities [1,14,15,16].

In the United States, when insurance coverage for gender-affirming care expanded with the Affordable Care Act (ACA), the Health Resources and Services Administration (HRSA), the Joint Commission, and the Institute of Medicine recommended routine collection of gender identity (GI) in healthcare settings in order to improve access to and quality of care. In 2014, the ACA’s Meaningful Use Stage 3 required Medicaid provider EHRs to have capacity to collect gender identity in a structured data field. By 2019, 53% (*n* = 360) of the 680 U.S. hospitals that participated in the Human Rights Campaign 2019 Health Equality Index reported collecting GI data, and 87% (*n* = 313) of those reported using the two-step method [17], capturing both GI and ASAB. Rush University Medical Center (RUMC), Rush Oak Park Hospital, and Rush Copley Medical Center in suburban Aurora are three of those 360 U.S. hospitals.

A 2019 study of 1367 U.S. Health Centers reporting to HRSA’s 2016 Uniform Data System analyzed the results to date and found that GI data was reported missing for 68% of patients [18]. This rate of uptake indicates major barriers to achieve the benefits ascribed to GI data collection and supports the contention that 40 years of insurance exclusions created a vast clinical training void for which GI data fields have not yet been able to compensate [19]. That is, patients and providers alike may hesitate to complete these fields as users mostly do not know how to interpret or communicate the information effectively [20,21,22,23]. The miscommunication or misuse of GI data in a clinical setting, due to a lack of provider training, may be a patient safety issue [24]. Another patient-centered reason for low penetration may have to do with the appropriateness of GI data collection during a particular encounter [25,26]. At the same time, secondary uses of clinical informatics and EHR data are the core of learning health systems science and offer a new, pragmatic research pathway for trans and nonbinary population health research [27].

### 1.3. Objective

The primary objective of this analysis is to assess markers of implementation effectiveness: the penetration and quality of GI-related data collection and, at the same time, to establish a 2020 benchmark for collection of gender identity, sex assigned at birth, as well as sexual orientation at this academic health center and for health systems across the United States. Secondarily, this analysis may provide insights into implementation barriers and facilitators as well as direction for gender identity, assigned sex at birth, and sexual orientation data quality standards.

## 2. Materials and Methods

### 2.1. Setting and Sample

Rush University Medical Center is a 727-bed hospital, tertiary care academic health center that serves Chicago and is located in the Near West Side. The Human Rights Campaign’s Health Equality Index has designated RUMC a national leader in the care of LGBTQ patients for 12 consecutive years [17]. RUMC uses Epic (Epic Systems Corporation, Verona, Wisconsin) for its electronic health record system. As with all EHR systems, Epic captures “sex” and refers to the sex associated with a patient’s insurance card or driver’s license. In addition, from approximately 2013 through 2019, RUMC captured SO/GI data in the social history section of the EHR. The social history section captures data related to the social determinants of health and structural vulnerabilities [28] that impact patient health outcomes (e.g., patient status with regard to housing instability, the legal system, intimate partner violence, substance misuse, sex work, etc.). This domain of the EHR is not accessible to the patient, is not considered essential medical information, and is therefore also easily bypassed or overlooked by providers.

**Figure 1:**
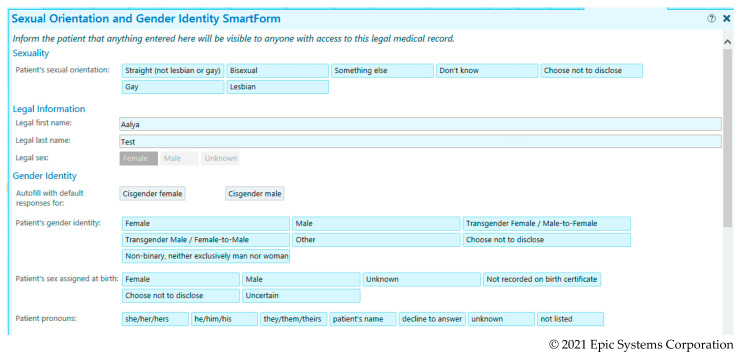
Epic Demographic Sexual Orientation (SO) and Gender Identity (GI) SmartForm (© 2021 Epic Systems Corporation) implemented for SO/GI data collection at Rush University Medical Center in 2020.

At the beginning of 2020, RUMC deployed the Epic sexual orientation (SO) and gender identity (GI) SmartForm, moving SO/GI data capture from social history to the demographics section of the EHR across the system. The demographics section has fields for ASAB, GI, and SO (see Figure 1); these are three of the SO/GI fields added to Epic in accordance with the 2015 Stage 3 Meaningful Use rules. Shifting the SO/GI data fields from social history to demographics enables a RUMC patient to access those fields and add or manually change their ASAB, GI, and/or SO once they are registered as a patient in the system. Although the legal name and legal sex appear in this tab, neither the patient nor provider is able to enter or change data in those fields, which are populated and affixed when a patient chart is established based on official insurance or identification. During an unplanned admission, the EHR workflow prompts the intake provider to complete these fields during intake or initial interview with a patient. However, the EHR does not have hard stops in this SmartForm, and the provider may bypass the fields due to time constraints, patient accessibility/unconsciousness, or discomfort with the questions. If the patient was already established as a patient from prior visits or admissions, the SO/GI fields may have been completed by providers known for gender-affirming care, or the patient.

The inclusion criteria for the quality improvement project were all unplanned adolescent and adult hospital patients (≥12 years of age) admitted between 1 January 2020 and 31 December 2020 (N = 49,314). This cohort includes all patients seen in the Emergency Department or admitted to the hospital, typically by way of the Emergency Department. The acute care setting and unplanned admissions are typically unrelated to planned, gender-affirming procedures with providers who are known for gender-affirming care. It is critical to assess the extent to which GI and ASAB data are collected in an acute care setting in order to evaluate patient experience, provider competencies, and to identify comorbidities and health outcomes associated with trans or nonbinary patients unrelated to gender-affirming procedures.

### 2.2. Definitions, Measures, and Analysis

Generally, cisgender refers to the alignment of one’s internal sense of self and the social expectations associated with one’s assigned sex at birth, whereas transgender or nonbinary is understood as having an internal sense of self that does not align with the social expectations associated with one’s assigned sex at birth. We defined a patient in the sample as cisgender if their GI and ASAB aligned (e.g., male and male) or, when the ASAB was missing or unknown, if their GI and legal sex aligned (e.g., female and female). We designated a patient transgender if their GI and ASAB fields differed in a meaningful way (e.g., male and female; other and female) or, if the ASAB field was unknown, we also deferred to the legal sex (e.g., legally female with male gender identity). While some trans persons may be hidden in cases counted as cisgender—for example, where ASAB is missing and GI and legal sex align—we conversely felt it was important to count patients as trans whose ASAB may have been missing but GI and legal sex did not align.

The genesis of the two-step question was to improve the accuracy of the census of trans patients. In particular, this method attempts to count trans people who may simply identify as men or women and do not explicitly identify as transgender [29]. In this analysis, if a GI field indicates “Other” or “Nonbinary,” we do not need to know their ASAB or legal sex to determine GI, as these two GIs indicate a gender that is explicitly outside the male/female gender binary. Therefore, if “Nonbinary” GI was selected with any other ASAB field or a null ASAB field, we counted the individual as nonbinary; similarly, “Other” GI and any ASAB field counted as transgender, “Other.” “Choose not to disclose” was the singular gender identity response option that was not counted as cis or trans but was not excluded from the cohort like the null values (i.e., empty cells).

Patient characteristics related to GI, ASAB, and SO were assessed and compared between legally female and legally male patients. No statistical tests were applied to these distributions due to imbalance and small cell sizes. Comparing RUMC trans/nonbinary patients vs. cisgender patients, Chi-Square tests were used to assess the demographic and health-related distributions (i.e., age ranges, race, ethnicity, payor, smoking status). Smoking status, collected in the social history section where SO/GI data were collected prior to 2020, was the only health outcome variable for comparison within the available dataset.

Data quality checks were conducted via manual chart review in the following three domains: (1) ostensible GI errors: we manually validated charts with combinations of legal sex, ASAB, and GI that suggested a clear misclassification (e.g., a legal male, assigned male at birth, with GI listed as trans male), and (2) a randomly selected 10% of the final trans sample, and (3) a randomly selected 10% of the 41 charts with errors in SO fields – where GI data appeared. Only in the case of GI misclassification errors did we check all cases and reclassify them for this analysis based on manual review. Notes, medication orders, procedure codes, and keywords were used to shift a GI status from transfeminine to transmasculine or in the other direction. To determine that a chart was that of a cisgender patient, a chart was absent of transgender-related keywords, procedures, and medications, and we also were able to find keywords, medication, conditions, or chosen names that might have led a provider staff to recode a chart from cisgender to trans, and/or we were able to find official documentation in the chart that contradicted the classification (e.g., a patient classified as “legal F” whose chart has a picture of their driver’s license and sex indicating “M”). For the quality check of the trans sample we evaluated for obvious misclassification errors or for any additional confirmation of trans status, but if no confirming data were found, we did not consider it a misclassification.

To estimate the trans and nonbinary patient population prevalence at RUMC, patient data were excluded if the ASAB and GI fields were empty. The study was deemed exempt from review as human subjects research and was approved as quality improvement by the Rush Institutional Review Board.

## 3. Results

### 3.1. Sample Characteristics

The 2020 cohort of unique patients, ages 12 years and older, who had unplanned hospital encounters, consists of 49,314 individuals, and 24% (*n* = 11,943) of them had GI data fields completed while 76% (*n* = 37,371) remain empty (see Table 1; see Table A1 in Appendix A for more granular GI-related data). Thirty-six patients with GI data had “Choose not to disclose” as the GI response option and were set aside as neither cisgender nor transgender/nonbinary. Nine patients had legal sex, ASAB, and GI fields misaligned in a way that suggested they might be transgender but did not make logical sense; manual validation determined that three are trans and six are cisgender (see Table 2). In addition to the nonbinary (1) and other (2) patients, seven patients had transmasculine or transfeminine gender identities and unknown ASAB. In sum, nearly 1% (*n* = 100) of unique patients with GI data had data that reflected a transgender or nonbinary status.

For sexual orientation, 80% (*n* = 39,862) of data fields were unpopulated (see Table 3). No patients’ SO data field contained the response option “Choose not to disclose.” Although these are not response options in Epic’s Demographic SO section, 13 patients had an SO data field indicating a “transgender” sexual orientation, and 28 patients had an SO data field indicating “neither exclusively male or female” as their sexual orientation. Excluding those 41 patients, 3.4% (*n* = 322) of all patients with populated SO data fields expressed a lesbian, gay, or bisexual sexual orientation. If we added the 28 nonbinary patients, who ostensibly have a non-heterosexual SO by virtue of being nonbinary, the prevalence of LGB patients would increase to 3.7%.

The distributions of trans/nonbinary patients differed from cisgender ones across all demographic domains except for ethnicity (i.e., Latinx, non-Latinx, or unknown). Of note, 62% of trans/nonbinary patients were between ages 18–34, compared to 30% of cisgender ones; 40% of cisgender patients were age 55 years or older compared to 8% of trans/nonbinary patients (see Table 4). Smoking status also differed with trans/nonbinary patient data indicating 22% currently smoke, compared to 10% of cisgender patients.

### 3.2. Data Quality

Nine patients had combinations of legal sex, ASAB, and GI data that were illegible in terms of a clear cisgender or transgender determination (see Table 2). In sum, after manual chart reviews, six patients were deemed cisgender and three transgender. Among one grouping, four patients were erroneously labeled legal female, assigned male at birth, and male GI (i.e., F/M/M). Three of these patients were cisgender, two women and one man. The man’s legal sex was miscoded as “F” and the women’s ASAB and GI were miscoded “M.” One of the cisgender women patients had her husband’s first name listed as her “preferred name,” and she takes a medication associated with transgender women, which may have led RUMC staff to misinterpret the patient’s chart as transgender and recode the patient as F/M/M. The fourth patient with data labeled F/M/M was determined to be transfeminine based on evidence of a legal feminine name, alternating misgendering and appropriate gendering by providers in the notes, along with transgender keywords in the notes, and gender-affirming hormones in the medication list.

Ten patients comprised the random 10% data quality check among the subsample of trans patients. Of these, five had additional information in the chart supporting trans status. For example, a patient deemed transgender male according to GI and ASAB data also had testosterone in his prescription medication list. Of the five patients with information in the chart supporting transgender status, three were transfeminine and two were transmasculine. Four patients had no supporting or contradicting information regarding transgender status. The tenth patient’s gender identity data had been changed between our initial data analysis and the subsequent data quality check from transgender to cisgender female. The procedural history for this patient is consistent with cisgender status.

Although sexual orientation data collection is not the primary focus here, we conducted a quality check on 10% of the 41 patients with gender identity-related data listed as their sexual orientation. As Figure 1 demonstrates, gender identities are not response options for sexual orientation. The chart review of one “transgender” case (i.e., patient Doe) and three “neither exclusively male or female,” revealed that all four charts had GI data in the EHR’s social history section, which-prior to 2020 when the SO/GI demographics SmartForm was deployed—was the primary place for SO/GI data collection. It appears that these data have been mismapped to sexual orientation, sometimes even overriding existing SO data that appears in either social history or the demographic field for sexual orientation. Three of four of these patient charts had lesbian, gay, and heterosexual sexual orientations in the SO field, and the fourth was empty. Instead, for these patients (*n* = 41), SmartPhrases related to gender identity like the Epic definition provided for nonbinary, “neither exclusively man or woman” (see Figure 1) were mapped to sexual orientation in our report.

## 4. Discussion

To our knowledge, this analysis is the first to benchmark implementation penetration and quality of gender identity-related data collection in U.S. academic health centers since the Meaningful Use incentivization for this data collection went into effect in 2014. Using structured data for gender identity and assigned sex at birth, 100 patients, or 0.84%, of the 2020 unplanned hospital patient cohort with GI data were identified as TGNB, in comparison to the estimated 0.5% of adults in Chicago in 2018, 0.51% of adults in Illinois in 2014, and 0.5–0.6% of adults in the United States in 2014 [30,31]. Compared to the cisgender hospital patients with gender ID data in 2020, the trans patients were disproportionately young, white, Medicaid insured, and had a higher prevalence of smoking. The disparities between the cis and trans samples indicate the critical importance of both SO/GI data collection and learning health systems science vis-à-vis equitable access to care, utilization outcomes, and health outcomes [32].

Our real-world data were too sparse and of limited quality to draw population-based conclusions. Although Rush is unique and a leader for its active and routine collection of SO/GI data [17], 76% of the EHR GI data fields remain unpopulated and highlight the need to address SO/GI data collection implementation appropriateness, penetration, and effectiveness more widely. Rush should continue to lead other systems and improve on the rate of SO/GI data collection and penetration with continued training efforts across hospital departments and at all levels within those departments. Among the populated fields of ASAB and GI as well as SO, the data quality requires improvement for more meaningful learning health systems research and clinical decision making. Widespread training regarding gender-affirming care and inclusive language and the development of SO/GI data quality standards will move U.S. healthcare closer to the goals of the Meaningful Use mandate. Patient Doe’s SO/GI data exemplify two key issues associated with our data quality checks: (1) the systemic error whereby some patients have GI data in their SO data fields indicates low SO/GI literacy among provider staff, in this case, who manage EHR data, and (2) the low literacy, alongside TGNB stigma, discrimination, and violence in U.S. healthcare settings may contribute to patient SO/GI disclosure choices.

Our QI study is not without limitations. In 2020, COVID-19 limited the number of non-COVID-19 related emergencies and unplanned admissions [33] biasing the sample compared to prior years. Selection bias also likely impacted our cohort whereby those patients with missing gender identity data are likely different from those patients who have GI data in their charts. Both provider and patient levels of literacy with concepts of gender identity are likely associated with whether or not a GI-related field is populated. Similarly, both the provider and patient’s comfort levels with discussion of gender identity and its relationship to health are likely associated with the presence or absence of GI-related data in the EHR. With unplanned hospital admissions, the severity and acuity of illness also matter and may influence provider perception of the appropriateness of SO/GI data collection in an acute care setting. Providers may have less time or concern to collect GI-related data from very sick patients.

## 5. Conclusions

Electronic health record data are not purely infrastructural [34]. Encounter data reflect moments of intense vulnerability for patients, particularly TGNB patients. These vulnerabilities are shaped not only by levels of patient health literacy and provider SO/GI literacy but also by the lack of durable legal protections in the U.S. that govern TGNB access to care, insurance coverage, and data privacy. Protections issued by executive order and rules, such as Section 1557 of the Affordable Care Act, that may change with each administration, limit acceptability and effectiveness of SO/GI data collection and disclosure in the United States [35,36]. Without more durable support and protections, SO/GI data collection will have a threshold that is fractional and fraught [27].

Rule-based algorithms and computable phenotypes that draw on a broad spectrum of structured data including diagnostic codes, procedure codes, medication orders, as well as a range of transgender and nonbinary-related keywords labeled in clinical notes have been generated to identify more comprehensive TGNB patient cohorts and associated health outcomes [23,37,38,39]. Machine learning and natural language processing (NLP) techniques may also operationalize critical information in clinical notes, rectifying current TGNB population health research gaps and relieving pressure on both patients and providers to disclose or collect SO/GI demographic data [40]. As with structured data collection, innovations to improve equity in gender-affirming care, research, and health outcomes will likely be more effective with a learning health systems science approach that includes ongoing provider training, patient education, and community and stakeholder engagement.

## Figures and Tables

**Table 1:** Gender identity data organized by assigned sex at birth (ASAB) for all Rush University Medical Center patients, 12 years and older, with unplanned hospital encounters in 2020 (N = 49,314).

	Assigned Sex at Birth				
Gender Identity	Female	Male	Nondisclosed	Unknown	Total
Man	28	3968		244	4240
Woman	7220	33	4	378	7635
Nonbinary	21	6		1	28
Other	1	1		2	4
Nondisclosed	9	7	11	9	36
Unknown	250	173	1	36,947	37,371
Total	7529	4188	16	37,581	49,314

**Table 2:** Legal sex, assigned sex at birth, and gender identity data (*n* = 9) that were illegible as cisgender or transgender status among the 2020 cohort of unique patients with unplanned admissions.

Error Labels		Corrected Labels			
Illegible Gender Identity		Cisgender		Transgender	
Legal Sex/ASAB/GI	*n* = 9	F/F/F	M/M/M	F/M/F	M/M/F
F/M/M	4	2	1	1	
M/M/TM	2				2
M/F/F	3	2	1		

**Table 3:** Sexual orientation data for all Rush University Medical Center patients, 12 years and older, with unplanned hospital encounters in 2020 (N = 49,314).

Sexual Orientation	Legal F	Legal M	TOTAL
Gay	11	111	122
Heterosexual	5696	3392	9088
Bisexual	67	37	104
Lesbian	96	0	96
Transgender *	11	2	13
Neither man nor woman *	19	9	28
Choose not to disclose	0	0	0
Null/missing	22,087	17,775	39,862
Total by legal sex	27,987	21,326	49,313

* Does not represent a response option to sexual orientation in EHR; represents mismapping of gender ID data into sexual orientation fields.

**Table 4:** Demographic traits and smoking status of unique hospital patients (≥age 12) in 2020, comparing patients with trans/NB GI data (*n* = 100) to patients with cisgender GI data (*n* = 11,806).

	Trans/Nonbinary		Cisgender			
	*n* = 100	%	*n* = 11,806	%	df, X^2^	*p*-Value
Age						
12–17	6	6%	246	2%	4, 66.79	<0.001
18–34	62	62%	3535	30%		
35–44	13	13%	1753	15%		
45–54	11	11%	1596	14%		
55+	8	8%	4676	40%		
Assigned Sex at Birth						
Male	40	40%	3969	34%	2, 7.49	0.02
Female	50	50%	7219	61%		
ASAB unknown	10	10%	618	5%		
Race						
Black	24	24%	4349	37%	5, 11.52	0.04
White	54	54%	4866	41%		
Other	14	14%	2029	17%		
Asian/Native Hawaiian	5	5%	382	3%		
Native American	1	1%	45	0%		
Declined to answer/unknown	2	2%	135	1%		
Ethnicity						
Hispanic/Latinx	16	16%	2553	22%	2, 5.19	0.07
Non-Hispanic/non-Latinx	82	82%	9186	78%		
Declined/unknown	2	2%	67	1%		
Payor						
Managed care/Medicaid	49	49%	4777	40%	3, 23.31	<0.001
Blue Cross/Private	38	38%	3684	31%		
Medicare	5	5%	2648	22%		
None documented	8	8%	452	4%		
Other payors	0	0%	204	2%		
Self-pay	0	0%	41	0%		
Smoking Status						
Current	22	22%	1213	10%	3, 43.42	<0.001
Former	18	18%	2967	25%		
Never	52	52%	7446	63%		
Null/not documented	8	8%	180	2%		

## Data Availability

Our dataset is derived from patient electronic health records, which include protected health information, and we cannot make it publicly available due to regulatory and legal restrictions imposed by Rush University Medical Center. Patient medical data is highly sensitive and, with quasi-identifiers such as race, ethnicity, and age, medical record data is reidentifiable when linked to other publicly available datasets. Should researchers who meet the criteria for access to this confidential data want to use our de-identified dataset to replicate the gender identity data collection implementation adoption and quality assessment, our Chief of Research Informatics at Rush (Casey Frankenberger) will serve as the point of contact outside our research team to inquire further about establishing a data use agreement and access to the patient data (cfranken@rush.edu).

## Appendix A

**Table A1:** Assigned sex at birth, gender identity, and legal sex EHR data for all Rush University Medical Center patients, 12 years and older, with unplanned hospital encounters in 2020 (*N* = 49,314).

	Legal F	Legal M	TOTAL
Assigned Sex			
Female	7511	18	7529
Male	23	4165	4188
Choose not to disclose	14	8	22
Not recorded on BC	2	0	2
Unknown/uncertain	4	4	8
Null/missing	20,433	17,131	37,564
Gender Identity			
Trans woman (AMAB)	8	8	16
Trans man (AFAB)	12	*3*	15
Cis woman (AFAB)	7220		7220
Cis man (AMAB)		3968	3968
Non-binary (AFAB)	19	*2*	21
Non-binary (AMAB)	0	6	6
Non-binary (unknown ASAB)	0	1	1
Woman (AMAB)	9	8	17
Man (AFAB)	4	*9*	13
Trans woman (unknown ASAB)		2	2
Trans man (unknown ASAB)		2	2
Woman (unknown ASAB)*	376		376
Man (unknown ASAB)*	3	239	242
Other (AFAB)	1		1
Other (AMAB)	1		1
Other (unknown ASAB)	2		2
Choose not to disclose	26	10	36
Null/missing	20,307	17,067	37,374
Legal Sex			
Female	27,987		27,987
Male		21,326	21,326
Unknown			1
TOTAL			49,314
